# Malaria knowledge, preventive actions, and treatment-seeking behavior among ethnic minorities in Ratanakiri Province, Cambodia: a community-based cross-sectional survey

**DOI:** 10.1186/s12889-018-6123-0

**Published:** 2018-10-26

**Authors:** Junko Yasuoka, Kimiyo Kikuchi, Keiko Nanishi, Po Ly, Boukheng Thavrin, Tsutomu Omatsu, Tetsuya Mizutani

**Affiliations:** 1 Research and Education Center for Prevention of Global Infectious Diseases of Animals, Tokyo University of Agriculture and Technology, 3-5-8 Saiwai-cho, Fuchu-shi, Tokyo, 183-8508 Japan; 2 Graduate Education and Research Training Program in Decision Science for Sustainable Society, Kyushu University, Motooka 744, Nishi-ku, Fukuoka-shi, Fukuoka, 819-0395 Japan; 3 Office of International Academic Affairs, Graduate School of Medicine and Faculty of Medicine, The University of Tokyo, 7-3-1 Hongo, Bunkyo-ku, Tokyo, 113-0033 Japan; 4 National center for Parasitology, Entomology & Malaria Control (CNM), Ministry of Health, #477 Betong Street (Corner St.92), Village Trapangsvay, Sanakat Phnom Penh Thmey, Khan Sen Sok, Phnom Penh, Cambodia

**Keywords:** Malaria, Knowledge, Preventive actions, Treatment-seeking behavior, Ethnic minorities, Cambodia

## Abstract

**Background:**

Malaria incidence has been steadily declining in Cambodia, where the government is aiming to eliminate malaria by 2025. Successful malaria elimination requires active engagement and participation of communities to recognize malaria symptoms and the development of prompt treatment-seeking behavior for early diagnosis and appropriate treatment. This study examined malaria knowledge, preventive actions, and treatment-seeking behavior among different groups of ethnic minorities and Khmer in Ratanakiri Province, Cambodia.

**Methods:**

Face-to-face interviews were conducted in December 2015, targeting 388 mothers with children under 2 years old, who belonged to ten ethnic minority groups or the Khmer group living in 62 rural villages in Ratanakiri. In addition to describing mothers’ knowledge and actions for malaria prevention, logistic regression analysis was performed to identify determinants of fever during the most recent pregnancy and among children under two.

**Results:**

Overall 388 mothers were identified for enrollment into the study of which 377 (97.2%) were included in analyses. The majority of mothers slept under bed nets at home (95.8%) and wore long-sleeved clothes (83.8%) for malaria prevention. However, knowledge of malaria was limited: 44.6% were aware of malaria symptoms, 40.6% knew the malaria transmission route precisely, and 29.2% knew of mosquito breeding places. Staying overnight at a farm hut was significantly associated with having fever during the most recent pregnancy (adjusted odds ratio [AOR] 2.008, 95% confidence interval [CI]: 1.215–3.321) and a child having fever (AOR 3.681, 95% CI 1.943–6.972). Mothers’ partaking in a variety of malaria preventive actions was protective against fever in children (AOR 0.292, 95% CI: 0.136–0.650). Among those who had fever during pregnancy, 39.4% did not seek treatment.

**Conclusion:**

Although the majority of mothers took malaria preventive actions, knowledge of malaria epidemiology and vector ecology and treatment-seeking behavior for fever were limited. Staying overnight at farm huts, regardless of the differences in socio-demographic and socio-cultural characteristics, was strongly associated with fever episodes during pregnancy and childhood. This study indicates the necessity of spreading accurate malaria knowledge, raising awareness of health risks related to agricultural practices, and promoting treatment-seeking behavior among ethnic minorities to strengthen their engagement in malaria elimination.

## Background

Malaria incidence and mortality have steadily declined in the Greater Mekong sub-region over the past decades [1]. In Cambodia, a marked decrease in *Plasmodium falciparum (P. falciparum)* has been observed since 2009; this coincides with the scale-up of the distribution of insecticide treated bed nets and the strengthening of Village Malaria Workers (VMWs) who provide early diagnosis and treatment of malaria for their villagers. The number of malaria cases has significantly decreased especially in western Cambodia (i.e. Pailin and Battambang Provinces) [2]. Given the successes observed in the malaria program, the Cambodian government is aiming to eliminate malaria by 2025 [3].

Although malaria transmission in Cambodia has been low recently, 21 of the 25 provinces are still endemic, and more than half of the population (approximately 8.6 million people) is still at risk of malaria. The risk of malaria transmission is the highest in the forest or forest fringe areas in the northeastern part of the country [4]. Although, four provinces in northeastern Cambodia (Rattanakiri, Mondulkiri, Steung Treng, and Kratie Provinces) have only 3% of the national population, they were reported to have had 43% and 25% of *falciparum* and *vivax* malaria cases in the country in 2013, respectively [2].

Ratanakiri Province consists mainly of rural, agricultural villages and has one of the lowest socioeconomic standards in the country [4]. Several studies have been conducted to examine the complexity of malaria epidemiology in the Province [5–7]. However, few studies have addressed the behavioral and social aspects of malaria. Therefore, little is known about malaria knowledge, preventive actions, and treatment-seeking behavior of ethnic minorities living in the Province.

In the elimination phase of malaria, complete termination of local transmission is required, especially in areas with foci of malaria transmission, which can remain as a source of infection [5]. In addition to appropriate infrastructure and sufficient resources at primary health care facilities, active engagement of communities to recognize malaria symptoms and take prompt treatment-seeking actions is crucial to reduce the reservoir of malaria parasites [8]. Therefore, communities need to be encouraged to take immediate actions to treat fever in order to promote early diagnosis and appropriate treatment of malaria [9]. Moreover, community knowledge, attitudes and practices should be examined to further promote proper malaria preventive actions and to discourage risk behavior among different ethnic minority groups.

The aim of this study was to examine malaria knowledge, preventive actions, and treatment-seeking behavior among ten ethnic minority groups and the Khmer group in Ratanakiri Province. Additionally, we aimed to identify risk factors for fever during the most recent pregnancy and among children under two. Results from this study will contribute to the development and implementation of future intervention programs to achieve malaria elimination in Cambodia.

## Methods

### Study design

This study was conducted as a baseline survey for an intervention study, which is currently carried out to integrate community-based malaria control and Maternal, Neonatal, and Child Health (MNCH) services in the Ratanakiri Province, Cambodia. Data were collected from December 23 to 30, 2015, through face-to-face interviews with mothers with children under 2 years of age living in 62 VMW villages.

#### Study site

This study was conducted in Ratanakiri Province in the north-eastern part of the country, which shares its borders with Lao People’s Democratic Republic (PDR) and Vietnam. Residents consist of 13 ethnic minority groups and the Khmer group, which is the major ethnic group in Cambodia. People generally live in villages which consist of 20–60 families and mainly engage in subsistence shifting agriculture, rice farming, and plantation of rubber, cashew, and cassava. As the majority of ethnic minority groups live on subsistence slash-and-burn farming and rice cultivation, many of them own farm huts (plot huts) near or inside the forest, where they stay to plant, guard, and harvest crops [10, 11].

Out of 27 health centers in Ratanakiri province, 12 that cover villages where Village Malaria Workers (VMWs) are engaged in malaria control activities, were included in this study (VMW villages). Seven out of the 12 health centers were then selected based on accessibility by vehicles or motorcycles throughout the year (purposive sampling). These included Andaung Meas, Chomrom Bay Sruk, Kachanh, Lumphat, Ochum, Oyadav, and Taveng. Of the 73 VMW villages serviced by the seven health centers, ten villages under the Ochum health center and one village under the Andaung Meas health center were excluded, considering the geographical difficulty in conducting a long-term intervention program scheduled after the survey, and absence of eligible interviewees at the time of the survey. Therefore, in total 62 VMW villages were selected for inclusion in this study.

### Data collection

#### Survey data

A semi-structured questionnaire was developed to conduct the face-to-face interviews. Most of the questions were drawn from existing questionnaires, including the Cambodia Demographic Health Survey 2014 [12] and questionnaires that were already used in Cambodia for previous studies on VMWs and mothers with children under five [13–15]. The questionnaire contained questions regarding a) mothers’ and their families’ socio-demographic characteristics; b) agricultural practices; c) history of fever and infectious diseases (malaria, diarrhea, and pneumonia, etc.); d) knowledge and actions for malaria prevention; e) treatment-seeking behavior of mothers for themselves and their children under two; f) mothers’ complications during pregnancy and childbirth (fever, anemia, bleeding, etc.); and g) children’s complications (fever, difficulty breathing, etc.). The questionnaire was developed in English, translated into Khmer by Cambodian health experts, and then verified by other Khmer health experts to ensure the accuracy of the translation.

Indices to measure mothers’ knowledge on malaria epidemiology and vector ecology and actions for malaria prevention and vector control were developed based on mothers’ answers to the survey questions, as described in previous publications [13, 16]. Knowledge indices measured to quantify understanding of malaria epidemiology and vector ecology included: malaria symptoms, malaria transmission, vector species, vector active time, vector development time, breeding places, and natural enemies. Regarding malaria symptoms, mothers were asked if the following are correct: stomach ache, diarrhoea, nausea, fever, and shivering. Choices given to answer malaria transmission included cough or sneeze, touching blood, touching utensils, sharing food, coming close to mosquitoes, and mosquito bites. Choices on mosquito genera and sex were given for vector species. For vector’s most active time, the following time periods were given, morning, afternoon, and dusk to dawn. Vector development time was asked by an open-ended question. Regarding vector breeding places, trees, on the ground, and water pools around houses, water pools in the forest were given. For natural enemies of the vector, four choices were given: dogs, birds, aquatic insects, and small fish. Correct answer for each item was given one point.

Action indices were developed to quantify different malaria preventive and vector control measures taken by mothers. Each measure that mothers undertook was given one or two points, according to its effectiveness and frequency [13, 16]. A maximum of two points were given to effective measures (always/most of the time = 2, sometimes/rarely =1, never = 0), including “come back home before dusk,” “wear long-sleeved shirts/pants”, “sleep under bed nets at home”, “refrain from going to the forest”, “bring hammock nets to the forest”, “fill in water pools” “burn trash around house”, “seal holes/cracks on walls/ceilings”, and “cover water jars/tanks”. A maximum of one point (always/most of the time/sometimes = 1, rarely/never = 0) was given to less effective measures including “kill mosquitoes by hands”, “use mosquito coils”, “spray house”, and “clear bush around house”. For a wrong measure “plant flowers/grasses around house”, 0 points (always/most of the time/sometimes/rarely = 0, never = 1) were given. The total points for five malaria preventive measures and nine vector control measures became index scores, ranging from 0 to 10 and 0 to 13, respectively. Using each index, study population was divided into tertiles, namely least active, active, and most active groups.

Seventeen surveyors visited each participant’s residence and conducted face-to-face interviews. Before conducting the interviews, malaria experts of the National Center for Parasitology, Entomology, and Malaria Control, Cambodia (CNM) and lead researchers conducted a one-day training for the surveyors to explain each question in the questionnaire and how to conduct the interviews.

We recruited all mothers with children under two residing in 62 VMW villages, who were available at the time of the survey, in order to capture malaria knowledge, preventive actions, and treatment-seeking behavior of mothers in the study site as accurately as possible. Although 388 mothers were interviewed, 11 were excluded from analysis because of missing data. In total, 377 mothers were included in the analysis.

#### GPS data collection and analysis

GPS data were collected to measure actual travel distance between the 62 villages and their nearest health centers. Four GPS data collectors traveled from each village to its nearest health center with Holux m-241 (a wireless GPS logger). GPS data were recorded every 5 seconds during each trip. Data were entered into Excel 2016 through the Holux exTour for Logger v2.1 software, and then into Arc Map 10.4, which was used to calculate the actual distance traveled.

### Data management and statistical analysis

All data were coded, entered into data analysis software, and double-checked by the authors to ensure accuracy. Data analysis was done by STATA version 14. Descriptive analyses were conducted to describe sociodemographic characteristics, knowledge and actions for malaria prevention, and treatment-seeking behavior for fever management among participants. Mothers were classified into socio-economic status quartiles based on household assets and housing characteristics determined using a principal component analysis [15, 17].

Basic descriptive statistics such as frequency and percentages were used to describe mothers with correct knowledge on malaria epidemiology and vector ecology as well as those who took each measure for malaria prevention and vector control. Knowledge and action indices were compared between mothers who had fever and those who did not have fever during the most recent pregnancy as well as between mothers with a child who had fever and those with a child who did not have fever during the last 3 months.

To identify determinants of fever during the most recent pregnancy and fever among their children less than 2 years of age, two multiple logistic regression analyses were conducted. Outcomes of these analyses were reported occurrence of fever during pregnancy and that of fever among children during the last 3 months. Also, the following independent variables were included: mother’s age, education, wealth quartiles, ethnicity, having other children or not, age and gender of the child, experience of child loss, whether water was treated prior to consumption, whether there was a toilet at home, whether the family owned livestock, indices to describe malaria preventive measures and vector control measures undertaken, and whether the mother had an agricultural practice which required staying overnight at a farm hut.

Of the sociodemographic characteristics investigated, literacy was excluded from the analysis because of multicollinearity with education (*r* = 0.89). Mother’s marital status and occupation were excluded because of the small sub-group sample sizes (most of the mothers were married and were farmers). Child age and child gender were also excluded from the analysis for fever during pregnancy because they were unknown factors during pregnancy. *P*-values < 0.05 were considered statistically significant.

## Results

Sociodemographic characteristics of 377 mothers with children under two living in 62 VMW villages are described in Table 1. The majority of the mothers (72.1%) were under thirty, most were married (97.6%, data not shown) and were farmers (91.3%, data not shown), and two thirds (68.2%) responded that they were illiterate. Most of the mothers were farmers (91.3%, data not shown). This study included mothers who belonged to the Khmer ethnic group (11.1%) and ten other ethnic minority groups, mainly Thompoun (23.3%), Charay (20.2%), and Kreung (15.4%). Approximately a tenth (11.1%) of the mothers had experienced stillbirth, and 13.0% had lost children in the past.

**Table 1:** Sociodemographic characteristics of the study population, stratified by the occurrence of fever during pregnancy and fever of children during the last 3 months (Total *n* = 377 in 62 villages)

Characteristics	Overall (*n* = 377)		Fever in pregnancy (*n* = 99)		*p*-value	Child had fever (*n* = 66)		*p*-value
	n	%	n	%		n	%	
Age								
< 20	80	21.2	26	26.3	0.165	16	24.2	0.622
20–29	192	50.9	50	50.5		32	48.5	
30–39	89	23.6	22	22.2		17	25.8	
40+	16	4.2	1	1.0		1	1.5	
Education								
None	251	66.6	65	65.7	0.795	45	68.2	0.975
Primary	98	26.0	28	28.3		17	25.8	
Secondary/above	28	7.4	6	6.1		4	6.1	
Literacy								
Cannot read at all	257	68.2	69	69.7	0.237	48	72.7	0.661
Able to read only part	100	26.5	28	28.3		16	24.2	
Able to read whole	20	5.3	2	2.0		2	3.0	
Husband’s education								
None	170	45.1	42	42.4	0.753	32	48.5	0.333
Primary	137	36.3	39	39.4		26	39.4	
Secondary and above	70	18.6	18	18.2		8	12.1	
Stay overnight at farm hut	188	49.9	62	62.6	0.003	48	72.7	< 0.001
Wealth quartiles								
Low	95	25.2	27	27.3	0.862	19	28.8	0.721
Lower middle	94	24.9	24	24.2		17	25.8	
Upper middle	94	24.9	26	26.3		13	19.7	
High	94	24.9	22	22.2		17	25.8	
Ethnicity								
Khmer	42	11.1	8	8.1	0.053	3	4.5	0.355
Thompoun	88	23.3	28	28.3		19	28.8	
Charay	76	20.2	14	14.1		14	21.2	
Kreung	58	15.4	22	22.2		10	15.2	
Other	113	30.0	27	27.3		20	30.3	
Number of children								
1	140	37.1	42	42.4	0.365	25	37.9	0.796
2	112	29.7	23	23.2		17	25.8	
3–4	99	26.3	26	26.3		18	27.3	
5+	26	6.9	8	8.1		6	9.1	
Child age (month)								
0–5	141	37.4	–	–	N/A	22	33.3	0.469
6–11	96	25.5	–	–		21	31.8	
12–17	73	19.4	–	–		14	21.2	
18–23	67	17.8	–	–		9	13.6	
Child gender (female)	181	48.0	–	–	N/A	36	54.5	0.242
Experience of stillbirth (Yes)	42	11.1	12	12.1	0.718	10	15.2	0.254
Experience of child loss (Yes)	49	13.0	13	13.1	0.963	13	19.7	0.075
Actual travel distance to health center (km)								
< 5.0	42	11.1	8	8.1	0.663	10	15.2	0.432
5.0 - 9.9	98	26.0	26	26.3		18	27.3	
10.0–14.9	132	35.0	38	38.4		18	27.3	
15.0 +	105	27.9	27	27.3		20	30.3	
Water treatment (Yes)	222	58.9	64	64.6	0.175	39	59.1	0.970
Have toilet (Yes)	73	19.4	19	19.2	0.960	15	22.7	0.446
Own livestock (Yes)	257	68.2	69	69.7	0.704	43	65.2	0.562

Marital status and occupation ommitted (Almost all are married and farmers)

Mothers’ knowledge and action for malaria prevention and vector control is summarized in Table 2. Less than half of the mothers were aware of malaria symptoms and transmission route (44.6% and 40.6%, respectively). The majority (78.0%) of the mothers recognized the time when mosquitoes are most active. Regarding other aspects of mosquito ecology, including breeding places, species, natural enemies, and time to develop from egg to adult stage, mothers’ knowledge was scarce (29.2%, 23.9%, 19.1%, and 3.4%, respectively). The majority of the mothers used bed nets at home (95.8%) and wore long-sleeved shirts/pants (83.8%) to prevent malaria. Approximately two thirds (68.7%) reported that they bring hammock nets to the forest. To control mosquitoes, approximately half of the mothers reported that they fill in stagnant water pools (50.1%), kill mosquitoes using their hands (48.5%), cover water jars/tanks (46.2%), burn trash around house (46.2%), and clear bush around house (40.8%).

**Table 2:** Knowledge and action for malaria prevention and vector control among the study population

		Overall	
		n	%
Knowledge of malaria epidemiology and vector ecology (correct answer)			
Malaria symptoms		168	44.6
Malaria transmission		153	40.6
Vector active time		294	78.0
Vector breeding places		110	29.2
Vector species		90	23.9
Natural enemies of vector		72	19.1
Vector development time		13	3.4
Actions for malaria prevention and vector control			
Malaria preventive measures (Always/Most of the time)	Sleep under bednets at home	361	95.8
	Wear long-sleeved shirts/pants	316	83.8
	Bring hammock nets to the forest	259	68.7
	Come back home before dusk	180	47.7
	Refrain from going to the forest	101	26.8
Vector control measures (Always/Most of the time)	Fill in water pools	189	50.1
	Kill mosquitoes by hands	183	48.5
	Cover water jars/tanks	174	46.2
	Burn trash around house	174	46.2
	Clear bush around house	154	40.8
	Spray house	72	19.1
	Use mosquito coils	57	15.1
	Seal holes/cracks on walls/ceilings	24	6.4
	Plant flowers/grasses around house	15	4.0

Indices to measure knowledge and action for malaria prevention and vector control were compared between mothers with and without fever during the most recent pregnancy and between mothers’ children with and without fever during the last 3 months (Table 3). Mothers who didn’t have fever during pregnancy had significantly greater knowledge about malaria symptoms compared to those who had fever during pregnancy (0.478 vs 0.354, *P* = 0.032). Mothers whose child did not have fever during the last 3 months had significantly greater knowledge about malaria symptoms (0.482 vs 0.273, *P* = 0.002), mosquito breeding places (0.585 vs 0.439, *P* = 0.030), and mosquitoes’ natural enemies (0.469 vs 0.258, P = 0.002) compared to mothers whose child had fever during the last 3 months. Additionally, a greater variety of malaria preventive measures were taken among mothers whose child didn’t have fever compared to those whose child had fever during the last 3 months (7.640 vs 6.636, *P* < 0.001).

**Table 3:** Indices to describe knowledge and action for malaria prevention and vector control

Indices and their items	Number of items in index	Maximum possible score	Reliability (Cronbach’s alpha)	Overall		Fever in pregnancy (*n* = 99)		No fever in pregnancy (*n* = 278)		*p*-value	Child had fever (*n* = 66)		Child no fever (*n* = 311)		*p*-value
				Mean	SD	Mean	SD	Mean	SD		Mean	SD	Mean	SD	
Knowledge															
Malaria symptom	5	1	0.837	0.446	0.498	0.354	0.481	0.478	0.500	0.032	0.273	0.449	0.482	0.500	0.002
Malaria transmission	6	1	0.877	0.406	0.492	0.364	0.483	0.421	0.495	0.321	0.303	0.463	0.428	0.496	0.061
Vector active time	1	1	N/A	0.780	0.415	0.778	0.418	0.781	0.415	0.954	0.742	0.441	0.788	0.410	0.421
Vector breeding places	4	1	0.691	0.560	0.497	0.525	0.502	0.572	0.496	0.423	0.439	0.500	0.585	0.493	0.030
Vector species	6	1	0.914	0.480	0.500	0.495	0.503	0.475	0.500	0.732	0.485	0.504	0.479	0.500	0.933
Vector natural enemies	4	1	0.731	0.432	0.496	0.364	0.483	0.457	0.499	0.109	0.258	0.441	0.469	0.500	0.002
Vector development time	1	1	N/A	0.034	0.183	0.051	0.220	0.029	0.167	0.310	0.030	0.173	0.035	0.185	0.838
Action															
Malaria preventive measures	5	10	0.649	7.464	2.114	7.182	2.164	7.565	2.090	0.122	6.636	2.291	7.640	2.035	< 0.001
Vector control measures	9	13	0.626	6.525	2.741	6.586	2.692	6.504	2.762	0.798	6.364	2.875	6.559	2.715	0.599

Factors associated with having fever during the most recent pregnancy and children having fever during the last 3 months are described in Table 4. Mothers who had an agricultural practice which required them to stay overnight at a farm hut were approximately twice as likely to have had fever during pregnancy, compared to those who don’t stay at a farm hut (adjusted odds ratio [AOR] 2.008, 95% confidence interval [CI]: 1.215–3.321). Children of mothers who stayed at a farm hut were 3.7 times more likely to have had fever during the last 3 months compared to those whose mothers don’t stay at a farm hut (AOR 3.681, 95% CI: 1.943–6.972). Additionally, children of mothers who were most active to prevent malaria were approximately 70% less likely to experience fever during the last 3 months compared to children with mothers who were less active in terms of malaria prevention (AOR 0.292, 95% CI: 0.136–0.650).

**Table 4:** Factors associated with the occurrence of fever: during pregnancy and child during the last 3 months

	Multiple logistic regression [Fever in pregnancy]			Multiple logistic regression [Fever of child]		
	AOR	95%CI		AOR	95%CI	
Stay overnight at farm hut	2.008	1.215	3.321**	3.681	1.943	6.972**
Malaria preventive measures (ref = least active)						
active	0.980	0.518	1.853	0.497	0.234	1.056
most active	0.736	0.386	1.405	0.292	0.136	0.650**
Vector control measures (ref = least active)						
active	0.888	0.485	1.626	1.297	0.623	2.702
most active	1.296	0.648	2.594	2.015	0.862	4.711
Age of mother						
< 20	1			1		
20–29	0.854	0.424	1.720	0.954	0.406	2.243
30–39	0.619	0.258	1.485	0.811	0.278	2.363
Education						
None	1			1		
Primary	1.270	0.698	2.312	1.352	0.657	2.780
Secondary and above	0.966	0.323	2.894	1.588	0.417	6.051
Wealth quartiles						
Low	1			1		
Lower middle	0.709	0.322	1.560	0.874	0.343	2.233
Upper middle	0.817	0.366	1.824	0.614	0.225	1.671
High	0.574	0.229	1.440	0.803	0.272	2.374
Ethnicity						
Khmer	1			1		
Thompoun	1.936	0.684	5.480	4.076	0.920	18.060
Charay	0.958	0.315	2.909	3.281	0.713	15.090
Kreung	2.158	0.751	6.199	2.112	0.455	9.809
Other	1.109	0.396	3.110	2.766	0.636	12.029
Have other children/sibling (Yes)	0.885	0.476	1.646	0.935	0.434	2.016
Age of child						
0–5	–	–	–	1		
6–11	–	–	–	1.456	0.701	3.024
12–17	–	–	–	1.154	0.512	2.601
18–23	–	–	–	0.713	0.285	1.783
Gender of child (Female)	–	–	–	1.317	0.725	2.394
Experience of child loss (Yes)	–	–	–	1.923	0.832	4.460
Water treatment (Yes)	1.351	0.772	2.366	0.951	0.484	1.867
Have toilet (Yes)	1.036	0.498	2.157	1.886	0.787	4.518
Own livestock (Yes)	1.445	0.738	2.829	0.939	0.419	2.107

*p < 0.05 **p < 0.01

Treatment-seeking behavior for fever management is summarized in Table 5. Among 377 study participants, 99 (26.3%) mothers had fever during the most recent pregnancy. Nearly half of them sought treatment from public providers: approximately 37.4% from the health center and 12.1% from VMWs. Approximately 40% of mothers did not seek treatment. Among those who sought treatment (*n* = 60), more than half (57.9%) sought treatment the same day or the day following the onset of fever. Approximately 60% were sure that they received a blood test for malaria diagnosis, and five of them were found to be positive (three *P. falciparum* cases and two *P. vivax* cases).

**Table 5:** Treatment-seeking behavior for fever during pregnancy and for children under two

	Fever during pregnancy (*n* = 99)		Children with fever in the last 3 months (*n* = 65)	
	n	%	n	%
Sought treatment from				
Hospital	2	2.0	3	4.5
Health center	37	37.4	31	47.0
Private clinic	5	5.1	9	13.6
Pharmacy	2	2.0	6	9.1
VMW	12	12.1	8	12.1
TBA	2	2.0	–	–
Didn’t seek treatment	39	39.4	9	13.6
Timing of treatment seeking (*n* = 60)			(*n* = 57)	
Same day	15	26.3	18	31.6
Next day	18	31.6	28	49.1
2 days later	15	26.3	7	12.3
3 days or more later	8	14.0	4	7.0
Don’t remember	4	7.0	–	–
Malaria diagnosis by blood test (*n* = 60)			(*n* = 57)	
Yes, *P.falciparum*	3	5.0	2	3.5
Yes, *P.vivax*	2	3.5	1	1.8
Yes, but not sure about result	10	17.5	3	5.3
Yes, negative	19	33.3	18	31.6
No, didn’t get tested	10	17.5	23	40.4
Don’t remember	16	28.1	10	17.5

Sixty-five mothers (17.2%) reported that their child had fever during the last 3 months. Nearly 60% of them sought treatment from public providers: about half (47.0%) from health centers and 12.1% from VMWs. About one out of seven mothers (13.6%) did not seek treatment. Out of mothers who sought treatment (*n* = 57), majority of them did so on the same day (31.6%) or the next day (49.1%) from the onset of fever. Less than half (42.2%) were sure that their child received blood test, and three reported positive results (two *P. falciparum* cases and one *P. vivax* case).

## Discussion

To our knowledge, this is the first study which reported knowledge and preventive actions for malaria and treatment-seeking behavior for fever among mothers with children under two in Ratanakiri Province, Cambodia. Although the majority of mothers used bed nets at home and wore long-sleeved clothes to prevent malaria, more than half of the mothers were not aware of malaria symptoms and transmission route. Staying overnight at a farm hut was significantly associated with having fever during pregnancy as well as a child having fever during the last 3 months. Mothers’ undertaking a variety of malaria preventive actions was found to be a protective factor against the development of fever among children. Our findings suggest that improvements are needed in treatment-seeking behavior as nearly half of the mothers did not seek treatment for fever during pregnancy, and one in seven mothers did not seek treatment when their child had a fever.

Major preventive actions against malaria were already undertaken among mothers in our study sites due to continuous efforts by the National Center for Parasitology, Entomology and Malaria Control, Cambodia. For example, the majority of mothers used bed nets at home and wore long-sleeved clothes to protect themselves from malaria. Also, two-thirds of the mothers reported that they brought hammock nets with them when they worked in the forests. However, actions to reduce the number of mosquitoes around their living environments were taken by fewer mothers. More than half of the mothers were not aware of malaria symptoms and its transmission route, and more than two-thirds of the mothers did not know where mosquitoes breed. To ensure malaria elimination, community engagement is the key; elements to increase community engagement include increased knowledge regarding the disease at individual and community levels [18]. Strengthening knowledge about malaria epidemiology and mosquito ecology can boost mothers’ actions for malaria prevention and vector control.

Staying overnight at a farm hut was identified as a significant risk factor for developing a fever during pregnancy and for children under two. It has been widely reported that agricultural practices are strongly associated with community health, including malaria incidence [19–21]. Recent studies using PCR diagnosis indicated that an overnight stay in a farm hut is a risk factor for malaria in Ratanakiri Province [5, 6]. Although this study focused on fever episodes, one of the symptoms of malaria, it is possible that malaria risk was increased by mothers’ staying overnight at a farm hut. This study provides behavioral and socio-cultural information regarding malaria as it includes data on different aspects of household characteristics and lifestyles, including educational background, ethnicity, wealth, family structure, travel distance to the nearest health centers, malaria knowledge, preventive actions, hygiene practices, and agricultural practices. These factors have not been considered in previous studies. Based on our findings, combined with those from two previous studies, it is strongly recommended that future educational and public health interventions for malaria elimination put high priority on farmers who stay overnight at farm huts in Ratanakiri Province.

In other Asian countries, the association between staying at farm huts and fever or malaria incidence is inconsistent. An entomological and epidemiological study conducted in north-west Thailand reported that malaria infection among farmers engaged in agricultural activities, including movement away from residences to farm huts for ploughing and planting for rice cultivation, was three times that of people living in the residential villages [22]. In Laos, staying overnight in a farm hut was not associated with an increased risk of malaria infection in a rural district, where farmers slept under insecticide-treated bed nets in the huts [23]. Therefore, local malaria epidemiology and a wide variety of socio-demographic and socio-cultural characteristics need to be taken into consideration when examining risk factors for local malaria incidence and/or fever.

We also found that pregnant women and mothers with small children in Ratanakiri Province need to be encouraged to take prompt actions to seek treatment for fevers. A striking finding was that nearly half of the mothers who had fever during pregnancy did not seek treatment. Low use of VMW services could be partly due to temporary suspension of the services in 2015 caused by the delay in payment process (Ly P., personal communication, December 21, 2017). The majority of pregnant woman and children, who received a blood test after running a fever, tested negative for malaria. However, it has been widely reported that pregnant women have an increased susceptibility to infection by *Plasmodia spp*. [24], and that malaria during pregnancy increases the risk of maternal death, miscarriage, stillbirth, and neonatal death [25, 26]. Additionally, early diagnosis and effective treatment of malaria is critical for communities to prevent further transmission and spread of the disease [27]. To ensure the elimination of malaria, communities at risk of becoming infected with malaria need to be aware of the importance of treatment-seeking behavior for fever in order to achieve complete and continuous termination of local transmission. Additionally, non-malarial fevers need either specific treatments or referral to health facilities for appropriate care to reduce maternal and child morbidity and mortality [28]. Therefore, it is strongly recommended that future educational interventions stress the importance of treatment-seeking behavior for fevers. Thus, the presence of community health workers such as VMWs in Cambodia, with whom villagers can promptly consult regarding their health issues, is important for malaria elimination.

The limitations of the study must be considered when interpreting these study findings. First, mothers were asked about health issues they experienced and the timing and frequency of their use of health services during their most recent pregnancy, which might have introduced recall bias. It could have resulted in underreporting of health issues and/or inaccurate reports of the number and timing of their health service use. Second, to examine preventive actions and treatment-seeking behavior, mothers’ self-reported data were used, which could have introduced courtesy bias. To minimize these biases and improve the accuracy of mothers’ self-reports about the timing and frequency of their use of health services, data were confirmed with a maternal health record book and a child health record book whenever available. Moreover, interviews were conducted by experienced and trained interviewers with on-site supervision. Third, sample size or power computation was not possible to be performed because of the lack of information on the number of mothers with children under two. Therefore, recruitment was performed to include as many mothers as possible in the study. However, we conducted a post hoc power analysis, and the sample size was determined to easily achieve a power of greater than 80% using the effect size we observed.

## Conclusions

To our knowledge, this was the first study to examine malaria knowledge, preventive actions, and treatment-seeking behavior of mothers who belonged to ten ethnic minority groups and the Khmer ethnic group, both of whom reside in remote, agricultural villages in Ratanakiri Province, Cambodia. This study revealed that even though the majority of mothers took malaria preventive measures, knowledge of malaria epidemiology and vector ecology and treatment-seeking behavior for fever were limited. The result indicates the importance of spreading accurate knowledge and promoting prompt treatment-seeking behavior to strengthen community engagement for malaria elimination. Furthermore, staying overnight at a farm hut, regardless of the differences in socio-demographic and socio-cultural characteristics, was strongly associated with fever during pregnancy and childhood. The findings of this study will contribute to the development and implementation of an effective community-based educational program to improve malaria knowledge, raise awareness of health risks related to agricultural practices, and promote treatment-seeking behavior, which will contribute to the achievement of malaria elimination in the region.

## Abbreviations

AOR: adjusted odds ratio
CI: 95% confidence interval
CNM: National Center for Parasitology, Entomology, and Malaria Control, Cambodia
MNCH: Maternal, Neonatal, and Child Health
*P. falciparum*: *Plasmodium falciparum*
PCR: polymerase chain reaction
VMW: village malaria workers
